# Trends in surgical aortic valve replacement in pre- and post-transcatheter aortic valve replacement eras at a structural heart center

**DOI:** 10.3389/fcvm.2023.1103760

**Published:** 2023-05-22

**Authors:** Elizabeth L. Norton, Alison F. Ward, Andy Tully, Bradley G. Leshnower, Robert A. Guyton, Gaetano Paone, William B. Keeling, Jeffrey S. Miller, Michael E. Halkos, Kendra J. Grubb

**Affiliations:** ^1^ Division of Cardiothoracic Surgery, Department of Surgery, Emory University, Atlanta, GA, United States; ^2^ Structural Heart and Valve Center, Emory University, Atlanta, GA, United States

**Keywords:** aortic valve, surgical aortic valve replacement, transcatheter aortic replacement, bioprosthetic aortic valve, aortic stenosis (AS), aortic insufficiency (AI), annular enlargement, short-term outcomes

## Abstract

**Background:**

The advent of transcatheter aortic valve replacement (TAVR) has directly impacted the lifelong management of patients with aortic valve disease. The U.S. Food and Drug Administration has approved TAVR for all surgical risk: prohibitive (2011), high (2012), intermediate (2016), and low (2019). Since then, TAVR volumes are increasing and surgical aortic valve replacements (SAVR) are decreasing. This study sought to evaluate trends in isolated SAVR in the pre- and post-TAVR eras.

**Methods:**

From January 2000 to June 2020, 3,861 isolated SAVRs were performed at a single academic quaternary care institution which participated in the early trials of TAVR beginning in 2007. A formal structural heart center was established in 2012 when TAVR became commercially available. Patients were divided into the pre-TAVR era (2000–2011, *n* = 2,426) and post-TAVR era (2012–2020, *n* = 1,435). Data from the institutional Society of Thoracic Surgeons National Database was analyzed.

**Results:**

The median age was 66 years, similar between groups. The post-TAVR group had a statistically higher rate of diabetes, hypertension, dyslipidemia, heart failure, more reoperative SAVR, and lower STS Predicted Risk of Mortality (PROM) (2.0% vs. 2.5%, *p* < 0.0001). There were more urgent/emergent/salvage SAVRs (38% vs. 24%) and fewer elective SAVRs (63% vs. 76%), (*p* < 0.0001) in the post-TAVR group. More bioprosthetic valves were implanted in the post-TAVR group (85% vs. 74%, *p* < 0.0001). Larger aortic valves were implanted (25 vs. 23 mm, *p* < 0.0001) and more annular enlargements were performed (5.9% vs. 1.6%, *p* < 0.0001) in the post-TAVR era. Postoperatively, the post-TAVR group had less blood product transfusion (49% vs. 58%, *p* < 0.0001), renal failure (1.4% vs. 4.3%, *p* < 0.0001), pneumonia (2.3% vs. 3.8%, *p* = 0.01), shorter lengths of stay, and lower in-hospital mortality (1.5% vs. 3.3%, *p* = 0.0007).

**Conclusion:**

The approval of TAVR changed the landscape of aortic valve disease management. At a quaternary academic cardiac surgery center with a well-established structural heart program, patients undergoing isolated SAVR in the post-TAVR era had lower STS PROM, more implantation of bioprosthetic valves, utilization of larger valves, annular enlargement, and lower in-hospital mortality. Isolated SAVR continues to be performed in the TAVR era with excellent outcomes. SAVR remains an essential tool in the lifetime management of aortic valve disease.

## Introduction

Aortic valve replacement remains one of the most commonly performed cardiac procedures; however, over the past decade there has been a shift in the management of aortic valve disease with the introduction of transcatheter aortic valve replacement (TAVR). TAVR was first approved and implemented in Europe, receiving Conformitè Europèenne (CE) Mark approval in 2007. However, it was not until November 2011 that TAVR was approved by the U.S. Food and Drug Administration (FDA) for use in patients with severe symptomatic aortic stenosis at prohibitive surgical risk and then in 2012 for patients considered high-risk for surgical aortic valve replacement (SAVR). Since 2019, TAVR has been approved for use in the United States for patients with severe symptomatic aortic stenosis at all levels of surgical risk (1). Although the overall volume of aortic valve replacements has increased since the approval of TAVR, stemming from dramatic expansion of TAVRs (13,723 total in 2011–2013 to 72,991 in 2019) (2), the annual number of SAVRs performed has decreased. Annual TAVR procedures now exceed all forms of SAVR (2, 3). These drastic changes in SAVR practice pattern have been associated with changes in patient population, surgical technique, and outcomes. However, the magnitude of these changes in contemporary SAVR remain underreported, especially at a practice-level. This study sought to examine trends in isolated SAVR and compare isolated SAVR in the pre-TAVR era (2000–2011) and the post-TAVR era (2012–2020). Clearer understanding of these trends may help to position SAVR more accurately as a key lifetime valve management therapy in cardiac surgery and structural heart practices.

## Materials and methods

### Study population

This retrospective review identified all adult (≥18 years) patients who underwent isolated SAVR at a single academic quaternary care institution from January 2000 to June 2020. Our institution participated in the investigational use of TAVR beginning in 2007 and established a structural heart center in 2012 when TAVR was approved for commercial use in the US. Of 3,861 isolated SAVRs, 63% (2,426/3,861) were performed in the pre-TAVR era (2000–2011) and 37% (1,435/3,861) were performed in the post-TAVR era (2012–2020). Aortic root replacements were excluded. Data were obtained from our institutional Society of Thoracic Surgeons (STS) Adult Cardiac Surgery Database and included pre-operative, operative, and post-operative data. Investigators utilized medical record review to supplement data collection. Since all data were deidentified, patient consent was waived by the institutional review board.

### Statistical analysis

Initial analysis provided descriptive information on the demographic, clinical, and surgical characteristics. Categorical variables were reported as count (percentage) and continuous variables were summarized by median (lower quartile, upper quartile). Categorical comparisons between pre- and post-TAVR groups were performed using chi-square tests or fisher exact tests as appropriate. Continuous data were compared using t-test or Wilcoxon rank sum tests as appropriate. Logistic regression was used to assess risk factors for in-hospital mortality adjusting for group, age, sex, renal failure on dialysis, prior stroke, prior myocardial infarction (MI), heart failure, cardiogenic shock, and prior cardiac surgery. All statistical calculations were performed using SAS 9.4 (SAS Institute, Cary, NC) and were considered significant at *p* < 0.05.

## Results

### Demographics and preoperative data

**Figure 1:**
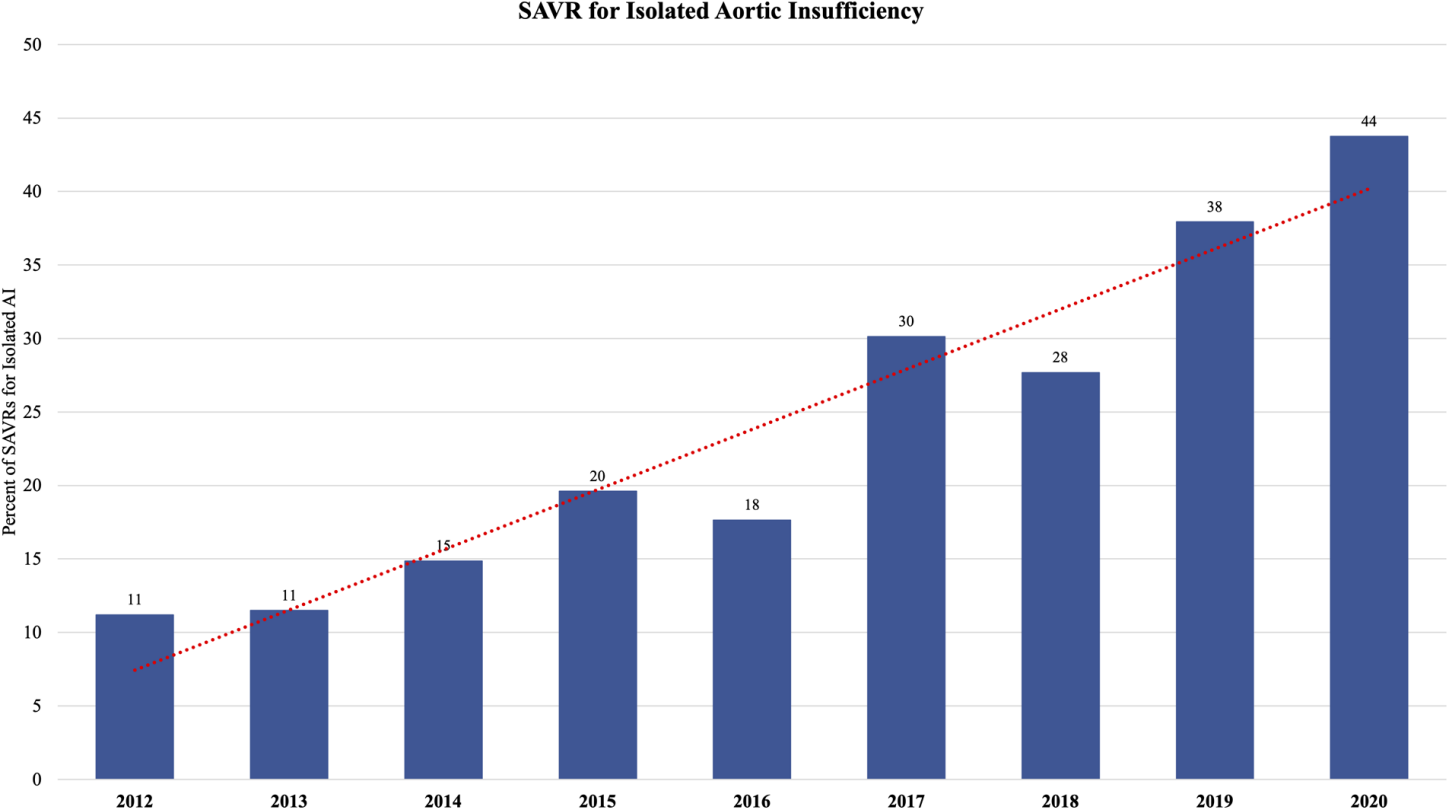
The proportion of patients undergoing isolated surgical aortic valve replacement (SAVR) for isolated aortic insufficiency (AI) increased in the post-TAVR era from 11% of isolated SAVRs in 2011 to 44% of isolated SAVRs in 2020.

**Figure 2:**
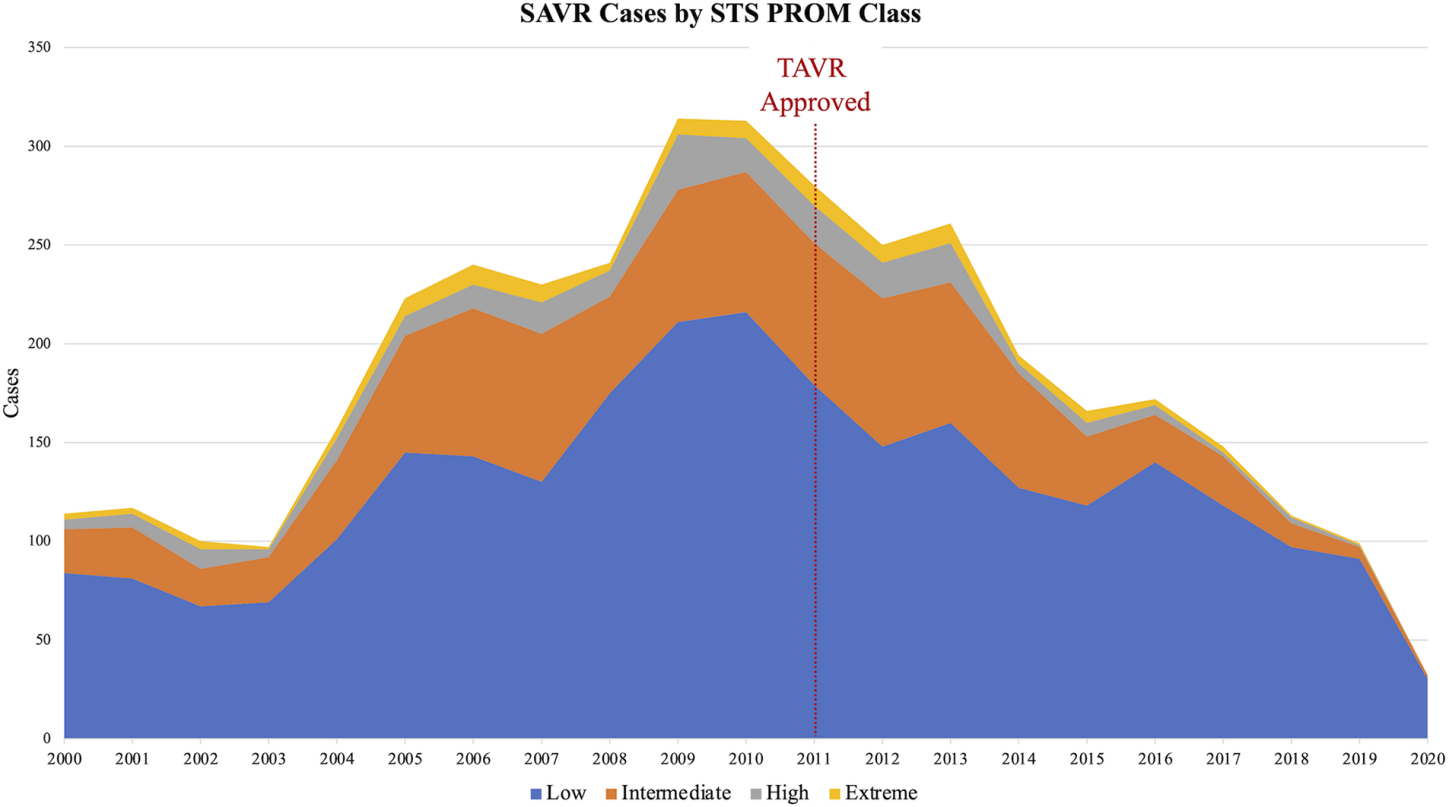
The society of thoracic surgeons (STS) predicted risk of mortality (PROM) of patients undergoing isolated SAVR decreased in the post-TAVR era. Low risk (blue) = STS PROM ≤3%; Intermediate risk (orange) = 3%< STS PROM ≤8%; High risk (gray) = 8%< STS PROM ≤15%; Extreme risk (yellow) = STS PROM >15%.

The median age of the entire cohort was 66 years and was similar between groups. Males comprised a majority (62%) of the cohort. The post-TAVR group had more diabetes (30% vs. 26%, *p* = 0.01), hypertension (86% vs. 77%, *p* < 0.0001), dyslipidemia (82% vs. 60%, *p* < 0.0001), heart failure (78% vs. 52%, *p* < 0.0001), and pervious cardiac interventions (34% vs. 31%, *p* = 0.02) compared to the pre-TAVR group. Reoperative aortic valve surgery was more common in the post-TAVR group, both previous aortic valve repair (2.1% vs. 1.0%, *p* = 0.007) and replacement (5.3% vs. 3.9%, *p* = 0.04). Regarding operative indication, preoperatively 80% of cases had aortic stenosis (AS) and 78% had some degree of aortic insufficiency (AI). Twenty percent of the entire cohort had moderate-to-severe AI without AS. In the post-TAVR era, there was an increasing percentage of patients with isolated moderate-to-severe AI undergoing isolated SAVR (from 11% in the 2012 to 44% in 2020) (Figure 1). The post-TAVR group had lower STS Predicted Risks of Mortality (PROM) (2.0% vs. 2.5%, *p* < 0.0001) (Table 1, Figure 2).

**Table 1:** Demographics.

	Total (*n* = 3,861)	Pre-TAVR (*n* = 2,426)	Post-TAVR (*n* = 1,435)	*p*-value
Age	66 (56, 75)	66 (55, 76)	66 (57, 75)	0.75
Sex, male	2,385 (62)	1,484 (61)	901 (63)	0.32
BMI	**28.1 (24.7, 32.4)**	**27.7 (24.4, 31.7)**	**28.8 (25.2, 33.1)**	**<0.0001**
BSA	**2.0 (1.8, 2.2)**	**2.0 (1.8, 2.2)**	**2.0 (1.9, 2.3)**	**<0.0001**
Diabetes	**1,070 (28)**	**638 (26)**	**432 (30)**	**0.01**
Hypertension	**3,096 (80)**	**1,859 (77)**	**1,237 (86)**	**<0.0001**
Dyslipidemia	**2,500 (69)**	**1,320 (60)**	**1,180 (82)**	**<0.0001**
Peripheral vascular disease	366 (10)	238 (11)	128 (8.9)	0.07
Prior stroke	319 (8.3)	195 (8.0)	124 (8.6)	0.51
Smoking history	**1,850 (48)**	**1,194 (49)**	**656 (46)**	**0.04**
Chronic lung disease	1,065 (27)	658 (27)	407 (28)	0.41
Renal failure on dialysis	161 (4.2)	107 (4.4)	54 (3.8)	0.33
Creatinine	**1.0 (0.8, 1.2)**	**1.0 (0.9, 1.2)**	**1.0 (0.8, 1.2)**	**<0.0001**
History of endocarditis	336 (9.1)	202 (9.0)	134 (9.3)	0.69
CHF	**2,382 (62)**	**1,268 (52)**	**1,114 (78)**	**<0.0001**
Prior MI	520 (13)	327 (13)	193 (13)	0.99
Cardiogenic shock	43 (1.1)	22 (0.9)	221 (1.5)	0.12
Previous cardiac intervention	**1,181 (32)**	**688 (31)**	**493 (34)**	**0.02**
Previous AV repair	**53 (1.4)**	**24 (1.0)**	**29 (2.1)**	**0.007**
Previous AV replacement	**164 (4.4)**	**91 (3.9)**	**73 (5.3)**	**0.04**
Aortic stenosis	2,854 (80)	1,704 (80)	1,150 (80)	0.96
Aortic insufficiency				**<0.0001**
None	796 (23)	511 (25)	285 (20)	
Trace/trivial	337 (9.7)	164 (8.0)	173 (12)	
Mild	767 (22)	394 (19)	373 (26)	
Moderate	612 (18)	363 (18)	249 (18)	
Severe	953 (28)	623 (30)	330 (23)	
Isolated AI	646 (20)	377 (20)	269 (19%)	0.53
Ejection fraction	58 (50, 60)	57.7 (50, 60)	58 (53, 63)	**0.006**
Predicted risk of mortality (PROM) (%)	**2.3 (1.3, 4.2)**	**2.5 (1.4, 4.5)**	**2.0 (1.1, 3.8)**	**<0.0001**
PROM risk category				**0.001**
Low-risk (PROM ≤3%)	**2,630 (68)**	**1,601 (66)**	**1,029 (71)**	
Intermediate-risk (3%<PROM≤8%)	**906 (23)**	**598 (25)**	**308 (21)**	
High-risk (8%<PROM≤15%)	**213 (5.5)**	**152 (6.3)**	**61 (4.3)**	
Extreme-risk (PROM>15%)	**112 (2.9)**	**75 (3.1)**	**37 (2.6)**	

Data presented as median (25%, 75%) for continuous data and *n* (%) for categorical data. AI, aortic insufficiency; AV, aortic valve; BMI, body mass index; BSA, body surface area; CHF, congestive heart failure in the past 2 weeks; MI, myocardial infarction; PROM, predicted risk of mortality; TAVR, transcatheter aortic valve replacement. *p*-value indicates the difference between pre-TAVR and post-TAVR groups.

Bolded text indicates statistical significance.

### Operative data

**Figure 3:**
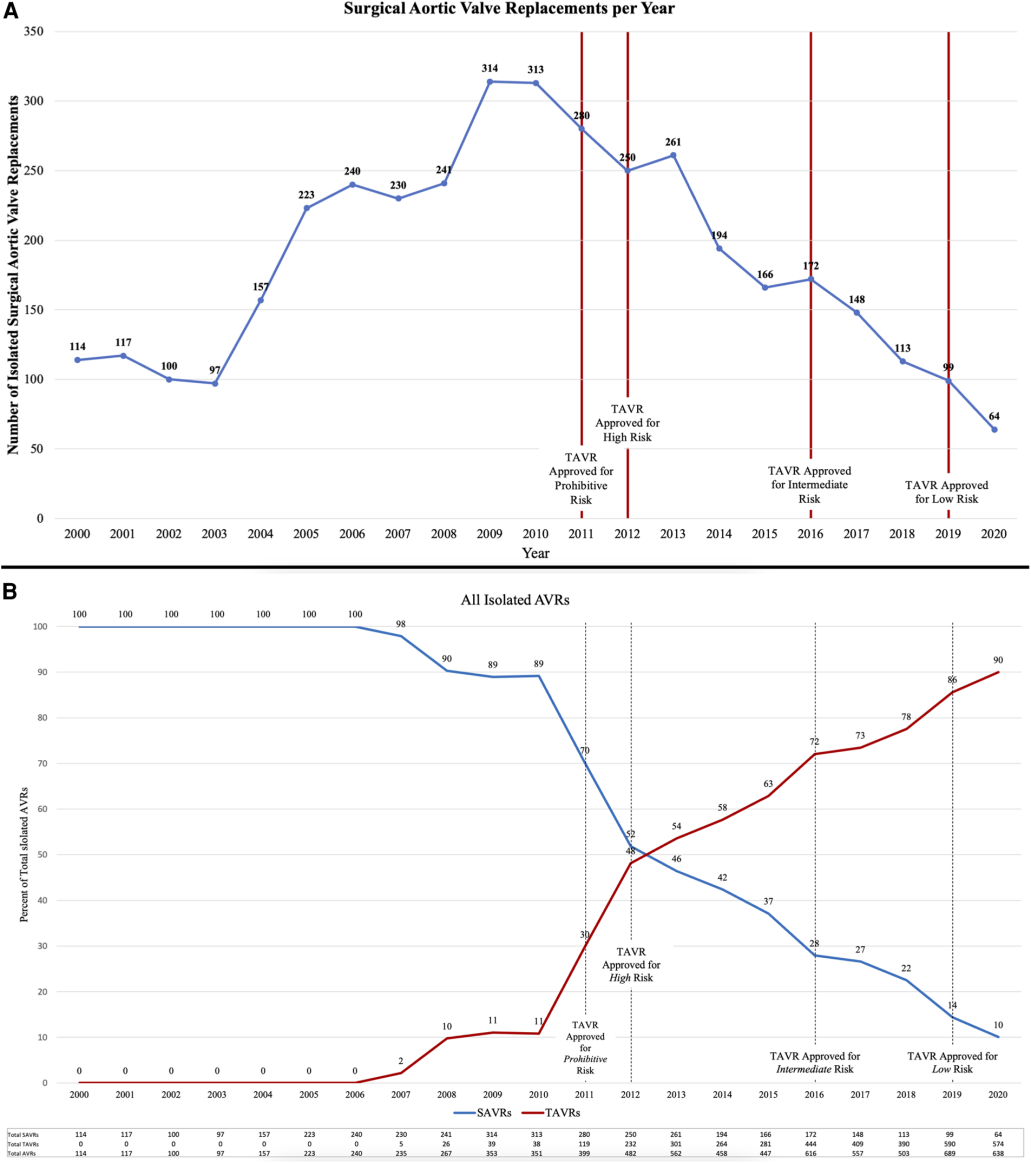
(**A**) The number of isolated surgical aortic valve replacements (SAVRs) performed annually decreased since the advent of transcatheter aortic valve replacement (TAVR) in 2011. (**B**) Over the years since TAVR approval, the number of TAVRs has increased while the number of SAVRs has decreased. TAVRs now make up a majority (90% in 2020) of isolated aortic valve replacements (AVRs) at a single institution with a structural heart center.

**Figure 4:**
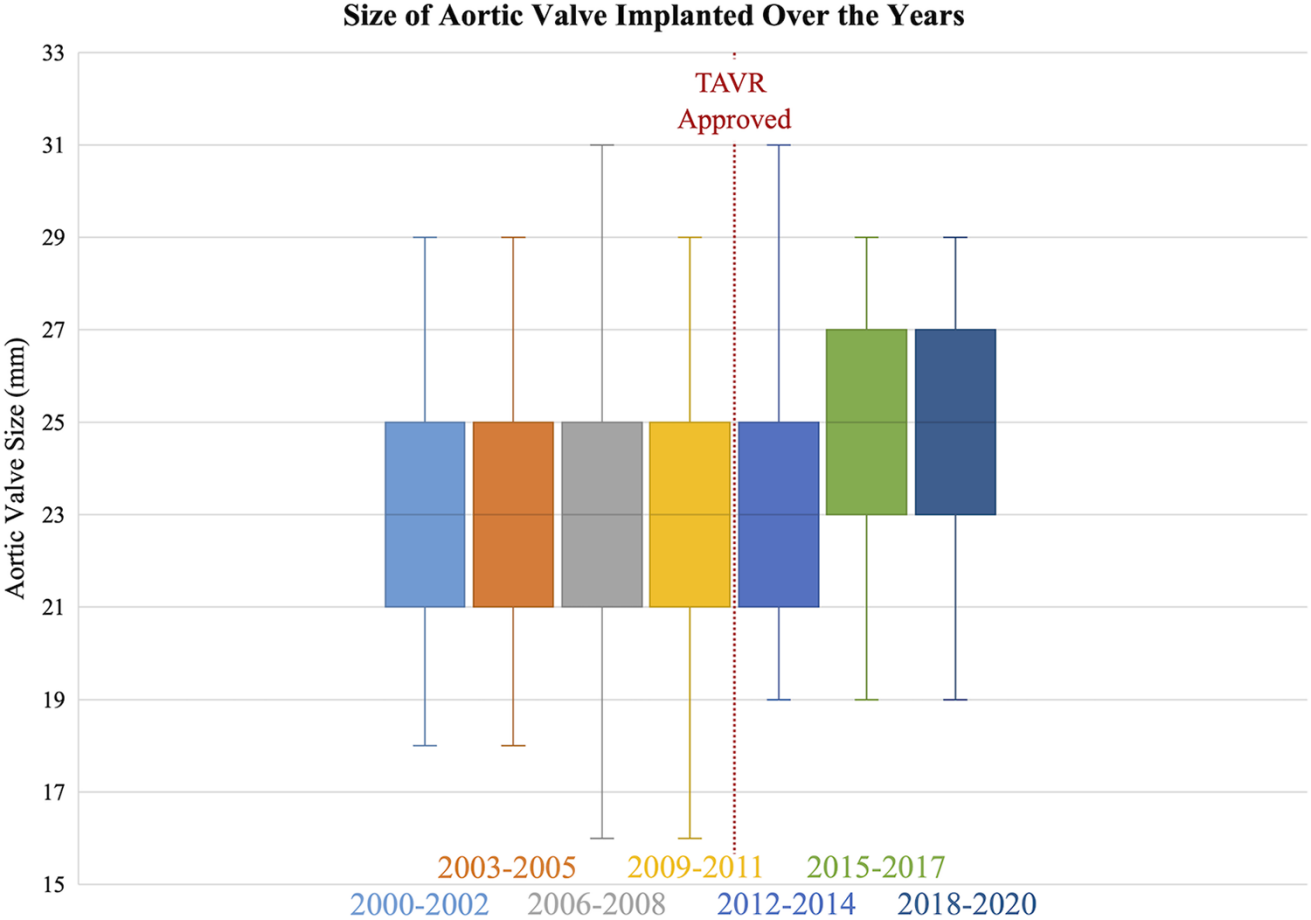
The median size of the surgical aortic valve implanted increased in the post-TAVR era (25 [23, 27] mm) compared to the pre-TAVR era (23 [21, 25] mm).

**Figure 5:**
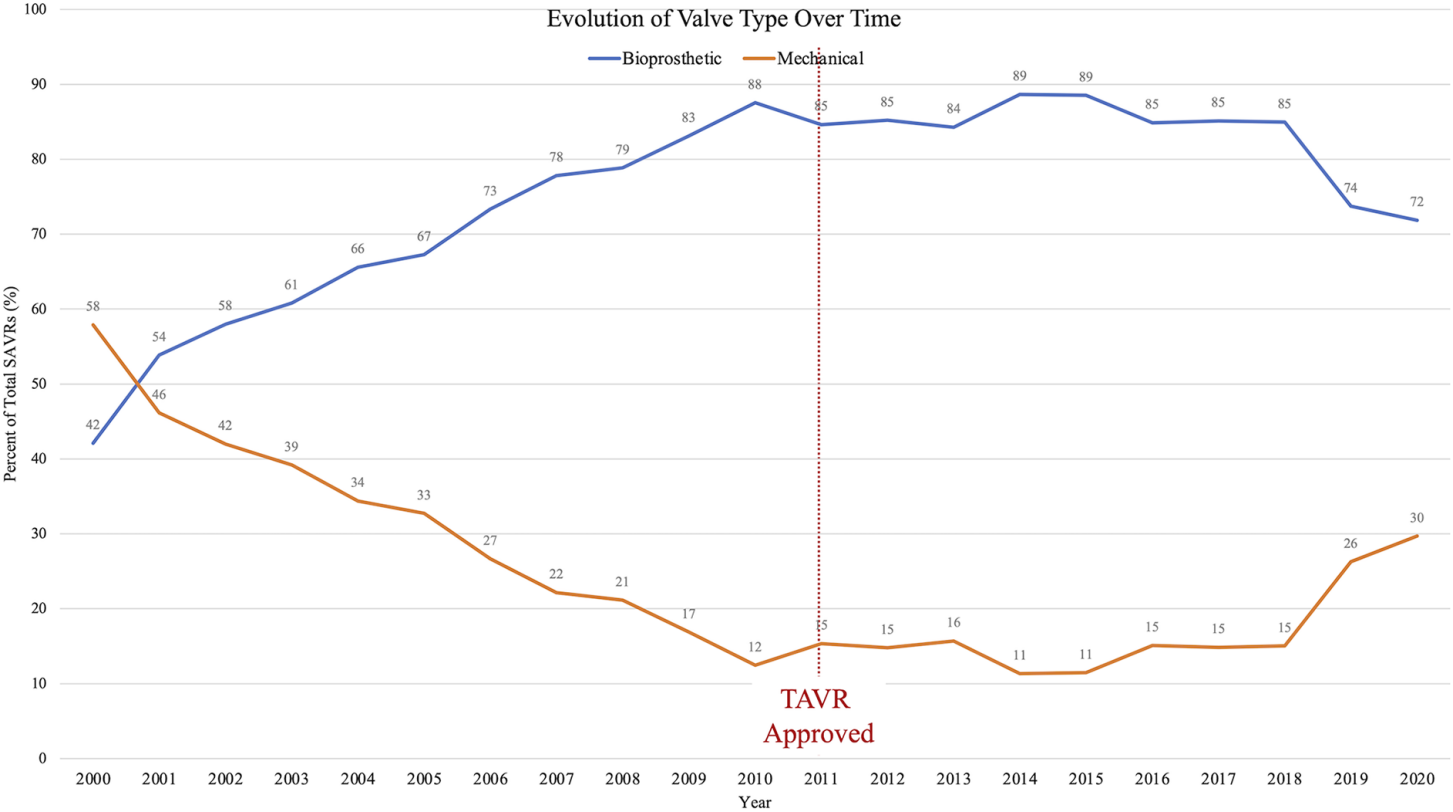
Over the past two decades, utilization of bioprosthetic heart valves has increased while mechanical valves decreased; however, this trend pre-dated the TAVR era. Since 2018, mechanical valve use is on the rise.

The number of SAVRs decreased in the post-TAVR era (Figure 3A) and decreased as the number of TAVRs at our institution increased (Figure 3B). Seventy-one percent of cases were elective SAVRs. The post-TAVR era had more urgent (36% vs. 23%), emergent (1.4% vs. 1.1%), salvage (0.1% vs. 0%) and less elective SAVRs (63% vs. 76%) than the pre-TAVR group (*p* < 0.0001). Eighty-one percent of patients underwent AVR via a full sternotomy; however, the post-TAVR era had more minimally invasive AVRs compared to the pre-TAVR era (19% vs. 15%, *p* = 0.005). SAVR was a redo-cardiac surgery in 17% of cases and was similar between pre- and post-TAVR groups (16% vs. 18%, *p* = 0.12). Among the reoperations, 55% (348/635) were prior valves, consisting mainly of prior aortic valves with 5 TAVR explants. Cardiopulmonary bypass time was similar between pre- and post-TAVR groups (107 vs. 106 min, *p* = 0.55); however, the post-TAVR group had slightly longer aortic cross-clamp times (82 vs. 80 min, *p* = 0.01) compared to the pre-TAVR group. In the entire cohort, the majority of aortic valves implanted were bioprosthetic (78%) with a median valve size of 23 mm. During the post-TAVR era, more bioprosthetic valves (85% vs. 74%, *p* < 0.0001) and larger valves (25 vs. 23 mm, *p* < 0.0001) were implanted compared to the pre-TAVR era (Figure 4). Among the bioprosthetic valves, the majority of valves implanted were stented valves across both the pre- and post-TAVR groups (76% vs. 78%, *p *= 0.40). Although more bioprosthetic valves were implanted in the post-TAVR era, the growing trend of increased implantation of bioprosthetic valves preceded TAVR approval (Figure 5). Annular enlargement was more commonly performed in the post-TAVR era (5.9% vs. 1.6%, *p* < 0.0001) (Table 2).

**Table 2:** Operative data.

	Total (*n* = 3,861)	Pre-TAVR (*n* = 2,426)	Post-TAVR (*n* = 1,435)	*p*-value
Reoperation	635 (17)	378 (16)	257 (18)	0.12
Number of prior operations				0.28
1	538 (88)	313 (89)	225 (88)	
2	55 (9.0)	29 (8.3)	26 (10)	
3	13 (2.1)	9 (2.6)	4 (1.6)	
4	2 (0.3)	0 (0)	2 (0.8)	
Status				**<0.0001**
Elective	2,732 (71)	1,835 (76)	897 (63)	
Urgent	1,082 (28)	565 (23)	517 (36)	
Emergent	46 (1.2)	26 (1.1)	20 (1.4)	
Salvage	1 (0.03)	0 (0)	1 (0.1)	
CPB time (min)	107 (92, 128)	107 (91, 129)	106 (92, 128)	0.55
Cross-clamp time (min)	**81 (68, 97)**	**80 (67, 96)**	**82 (70, 98)**	**0.01**
Aortic valve type				
Bioprosthetic	3,008 (78)	1,798 (74)	1,216 (85)	**<0.0001**
Stented	2,290 (77)	1,345 (76)	945 (78)	0.40
Stentless	688 (23)	417 (24)	271 (22)	0.40
Mechanical	847 (22)	628 (26)	219 (15)	**<0.0001**
Aortic valve size	**23 (21, 25)**	**23 (21, 25)**	**25 (23, 27)**	**<0.0001**
Annular enlargement	**116 (3.5)**	**31 (1.6)**	**85 (5.9)**	**<0.0001**
IABP	**180 (4.7)**	**100 (4.1)**	**80 (5.6)**	**0.04**

Data presented as median (25%, 75%) for continuous data and *n* (%) for categorical data. CPB, cardiopulmonary bypass; IABP, intra-aortic balloon pump; TAVR, transcatheter aortic valve replacement. *p*-value indicates the difference between pre-TAVR and post-TAVR groups.

### Postoperative outcomes

The post-TAVR group received less blood product transfusion than the pre-TAVR group (49% vs. 58%, *p* < 0.0001). Among those requiring transfusion, the post-TAVR group required less packed red blood cells (2 [1, 4] vs. 2 [2, 4] units, *p* = 0.0002). The post-TAVR group had less postoperative renal failure (1.4% vs. 4.3%, *p* < 0.0001) but similar rates of renal failure requiring dialysis (1.3% vs. 2.1%, *p* = 0.08). The post-TAVR groups had less pneumonia (2.3% vs. 3.8%, *p* = 0.01) but longer time in the intensive care unit (48 vs. 40 h, *p* < 0.0001). Despite longer time in the ICU, the post-TAVR group had a shorter postoperative length of stay (6 [5, 8] vs. 6 [5, 9] days, *p* = 0.046). Mortality improved across the study period; the post-TAVR group had lower in-hospital mortality (1.5% vs. 3.3%, *p* = 0.0007) than the pre-TAVR group (Table 3).

**Table 3:** Postoperative outcomes.

	Total (*n* = 3,861)	Pre-TAVR (*n* = 2,426)	Post-TAVR (*n* = 1,435)	*p*-value
Blood product	**1,981 (55)**	**1,280 (58)**	**701 (49)**	**<0.0001**
PRBCs (units)	**2.0 (1.0, 4.0)**	**2.0 (2.0, 4.0)**	**2.0 (1.0, 4.0)**	**0.0002**
Reoperation for bleeding	144 (3.7)	94 (3.9)	50 (3.5)	0.54
Reoperation for valve dysfunction	10 (0.3)	4 (0.2)	6 (0.4)	0.20
Renal failure	**120 (3.2)**	**100 (4.3)**	**20 (1.4)**	**<0.0001**
Requiring dialysis	68 (1.8)	49 (2.1)	19 (1.3)	0.08
Dialysis at discharge	7 (0.2)	2 (0.1)	5 (0.4)	0.07
Time in ICU (hours)	**46 (25, 79)**	**40 (24, 46)**	**48 (27, 88)**	**<0.0001**
Prolonged ventilation	502 (12)	334 (14)	168 (12)	0.07
Pneumonia	**125 (3.2)**	**92 (3.8)**	**33 (2.3)**	**0.01**
Postoperative LOS (days)	**6 (5, 9)**	**6 (5, 9)**	**6 (5, 8)**	**0.046**
Total LOS (days)	**7 (5, 11)**	**7 (5, 12)**	**7 (5, 10)**	**0.002**
In-hospital mortality	**100 (2.6)**	**79 (3.3)**	**21 (1.5)**	**0.0007**
30-day readmission	366 (11)	231 (11)	135 (11)	0.60

Data presented as median (25%, 75%) for continuous data and *n* (%) for categorical data. LOS, length of stay; PRBC, packed red blood cells; TAVR, transcatheter aortic valve replacement. *p*-value indicates the difference between pre-TAVR and post-TAVR groups.

Logistic regression demonstrated age (OR = 1.04 [95% CI: 1.02, 1.06], *p* < 0.0001), prior stroke (OR = 2.06, [95% CI: 1.17, 3.62], *p* = 0.01), renal failure on dialysis (OR = 5.86, [95% CI: 3.18, 10.8], *p* < 0.0001), heart failure (OR = 3.06, [95% CI:1.73, 5.44] *p* = 0.0001), cardiogenic shock (OR = 7.47, [95% CI: 2.82, 19.8], *p* < 0.0001), and pre-TAVR era (OR = 2.74, [95% CI: 1.63, 4.61), *p* = 0.0001) as independent predictors of in-hospital mortality (Table 4). Thirty-day readmission was 11% and statistically similar between groups.

**Table 4:** Risk factors for in-hospital mortality.

	Odds ratio [95% CI]	*p*-value
Pre-TAVR era	**2.74 [1.63, 4.61]**	**0.0001**
Age	**1.04 [1.02, 1.06]**	**<0.0001**
Sex	1.17 [0.75, 1.81]	0.49
Prior MI	1.06 [0.61, 1.84]	0.83
Cardiogenic shock	**7.47 [2.82, 19.8]**	**<0.0001**
Prior stroke	**2.06 [1.17, 3.62]**	**0.01**
Renal failure on dialysis	**5.86 [3.18, 10.8]**	**<0.0001**
CHF	**3.06 [1.73, 5.44]**	**0.0001**
Prior cardiac surgery	1.36 [0.81, 2.31]	0.25

CHF, congestive heart failure in the past 2 weeks prior to surgery; CI, confidence interval; MI, myocardial infarction; TAVR, transcatheter aortic valve replacement.

## Discussion

**Figure 6:**
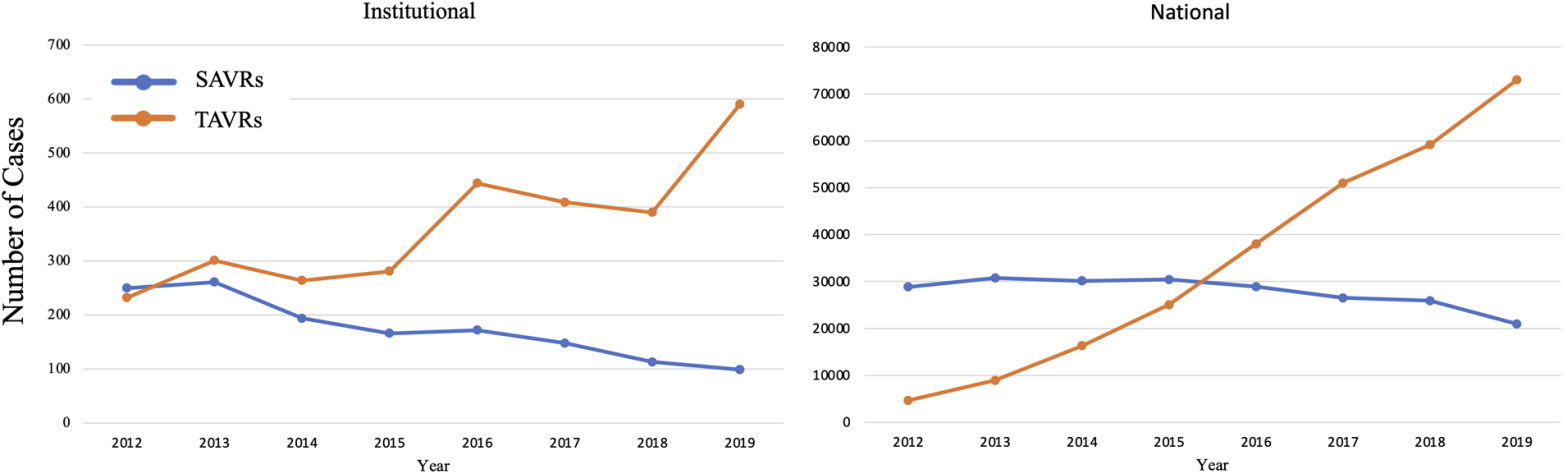
Since the approval of TAVR in 2011, the number of TAVRs have been increasing while SAVRs have been decreasing. This trend at a quaternary heart center is similar to that seen nationally.

At a high-volume quaternary cardiac surgery center with a well-established structural heart program, isolated SAVR cases/year have decreased in the post-TAVR era in a similar fashion to what has been reported nationally (Figure 6). Comparing the pre- and post-TAVR eras, patients undergoing isolated SAVR in the post-TAVR era had lower STS PROM, more implantation of bioprosthetic and larger valves with more annular enlargement procedures, and better postoperative outcomes including in-hospital mortality (1.5% vs. 3.3%). Across both eras, SAVR continues to be safe and associated with a low overall morbidity and mortality.

Over the past decade, multiple randomized control trials have shown the safety and efficacy of TAVR with 30-day mortality ranging from 0.5% to 3.9% (4–9). Since 2011, TAVR volume has grown annually, exceeding all forms of SAVR in 2019 (72,991 vs. 57,626) (2). Concomitantly, the patient population undergoing SAVR has evolved. This study found that patients undergoing SAVR in the post-TAVR era had lower STS PROM compared to the pre-TAVR era with 71% of patients undergoing SAVR in the post-TAVR era having a STS PROM ≤3% and 92% ≤8%, similar to the statewide experience in Michigan (10). This change may signify appropriate risk stratification under a Heart Team model and was likely due to more high-risk patients undergoing SAVR in the pre-TAVR era who are now undergoing TAVR. In the post-TAVR group, 6.8% (98/1,435) of patients were high or extreme risk (STS PROM >8%). Among the 98 high or extreme risk surgical patients in the post-TAVR era, 34% (33/98) had endocarditis and thus were not candidates for TAVR. All patients were evaluated by a multidisciplinary heart team and underwent pre-procedure CT assessment for anatomic candidacy. Common reasons patients were declined for TAVR include risk of coronary occlusion due to low coronary ostia heights or sinus sequestration due to long leaflet height and low sinotubular junction height. Patients with small surgical valves and likely pre-existing patient-prosthesis mismatch would also be referred for surgery. In addition, VIV TAVR was approved in 2015 for only failed surgical bioprosthetic valves in patients deemed high risk for open surgery (11).

These findings were consistent with a Society of Thoracic Surgeons-American College of Cardiology Transcatheter Valve Therapy Registry review by Carroll et al. (2), where the median STS PROM of patients undergoing TAVR from 2011 to 2019 was 5.2% higher than those undergoing SAVR in the post-TAVR era in this study (2.0%). Over time the STS PROM of patients undergoing TAVR decreased, from 6.9% before 2014 to 4.4% in 2019 (2), likely attributable to the broadening of indications for TAVR to intermediate- and low-risk patients. Similarly, in-hospital mortality of patients undergoing TAVR decreased from 5.4% before 2014 to 1.3% in 2019 (2). Therefore, in the TAVR era, mortality following aortic valve replacement (TAVR and SAVR) has significantly improved, <2% overall [TAVR 2.0% (2) and in this study SAVR 1.5%]. This underscores the evolution of aortic valve disease management over the last decade- with higher risk patients appropriately undergoing TAVR and lower risk patients undergoing SAVR.

TAVR has not only altered the patient population undergoing SAVR but also the operation itself, specifically the incision as well as the type and size of valve to be implanted. With the increased patient interest in minimally invasive surgery over the years, new technologies, and transcatheter techniques cardiac surgery and aortic valve replacements have evolved. Although the majority of SAVRs remain to be done via sternotomy, less invasive techniques such as mini-sternotomy or mini-thoracotomy are being employed. In this study, the post-TAVR era saw an increase in minimally invasive SAVRs (19% vs. 15%, *p* = 0.005). Minimally invasive AVRs have been shown to decrease length of stay, but have similar mortality and long-term results (12). However, it is important to recognize that patients undergoing minimally invasive AVR have less comorbidities and comprise a select cohort of AVR patients.

Now, bioprosthetic valves are the most commonly implanted, more than 85% (13). Lifetime valve management strategies now demand balancing considerations including valve durability, risk of reoperation, risk of long-term anticoagulation, and with the approval of valve-in-valve (ViV) TAVR in 2015 the possibility of a ViV TAVR following SAVR. Other studies (13) have shown an increased utilization of bioprosthetic valves in the current era as does this study in which 85% of valves implanted in the post-TAVR era were bioprosthetic compared to 74%. Although similar to mechanical valves in terms of survival (14), bioprosthetic valves are limited by durability. Durability and freedom from structural valve deterioration depend on a variety of factors including the valve model, age at valve implant, severity of valvular disease, and cardiac remodeling, but on average most valves are expected to last 10–20 years (15, 16). Therefore, younger patients often require more than one aortic valve intervention. Multiple combinations exist utilizing SAVR and TAVR for younger patients, yet no consensus exists for an optimal strategy (17). Interestingly, in this study, following a steady decline in the use of mechanical valves, since 2018 there was an increase (Figure 5). The renewed use of mechanical valves may represent a shift away from very young patients receiving biologic valves with the promise of future ViV TAVR as we have learned more about the feasibility of this strategy. However, in the era of TAVR, SAVR remains a valuable first option with low risk of morbidity and mortality as demonstrated by decreased postoperative complications and low mortality rate in this study. ViV TAVR could be an option for younger patients who underwent prior SAVR with a bioprosthesis; however, the SAVR valve must be a large enough valve (optimally ≥25 mm) to facilitate future ViV TAVR (18, 19).

Implantation of an appropriately sized valve for the patient is imperative; the inner diameter of the prosthetic valve should match the patient's native annulus (basal ring) (20) and patient-prosthesis mismatch (PPM) during the initial SAVR and future ViV TAVR should be considered. Patient-prosthesis mismatch occurs when the effective orifice area (EOA) of the implanted prosthetic valve is too small for the patient's body size (21) and contributes to structural valve deterioration (22, 23), associated with worse long-term survival (24). Aalaei-Andabili et al. (25) found the incidence of PPM was almost double following SAVR compared to TAVR (54% vs. 29%, *p* < 0.001), especially among patients receiving a valve size ≤23 mm (SAVR, 65% vs. TAVR, 48%, *p* = 0.048). To avoid PPM in the initial SAVR operation, aortic annular/root enlargement can be performed; as evident in this study where more annular enlargement procedures were performed and the median valve size of implanted valves increased to 25 mm in the post-TAVR era. Notably our patient population had more obesity and larger body surface areas, thus placing the patients at a higher risk of PPM.

SAVR provides the opportunity to up-size the valve, particularly with newer aortic root enlargement techniques. For example, the Y incision and roof patch technique described by Yang et al. (26–29) which can increase the implanted valve by five valve sizes to provide patients optimize hemodynamics, longevity of the bioprosthesis, and a better platform for future ViV TAVR. Although aortic root enlargement has not been widely adopted, it is now more commonly performed in the TAVR era. In this study, root enlargement increased from 1.6% in the pre-TAVR era to 5.9% in the post-TAVR era and the median valve size implanted increased from 23 to 25 mm. However, our operative mortality decreased by 50% (1.5% from 3.3%) during this timeframe. Shih et al. (30) and Coutinho et al. (31) found that adding an aortic root enlargement procedure to a SAVR does not increase surgical risk, but this is not yet a consensus. In one multi-institutional study utilizing the STS database (32), there was an increased risk of renal failure and operative mortality with aortic root enlargement. However, in that study the annular enlargement group received statistically smaller valves than the no annular enlargement group (22.7 vs. 23.2 mm, *p* < 0.001) with fewer patients in the annular enlargement group receiving a valve ≥23 mm (55% vs. 64%); additionally, the groups were different comparing patients with a small annulus (19–21 mm) needing aortic root enlargement to patients with a large annulus (≤23 mm) not needing aortic root enlargement.

In the current post-TAVR era, shared decision making for lifetime aortic valve disease management will lead many patients to choose TAVR, although the long-term outcomes of TAVR remain unknown. TAVR is a good option for many, in particular those at high surgical risk or older patients with a shorter life expectancy. For younger patients with life expectancy >10 years, especially low risk patients, SAVR remains a reasonable option with decades of data to support durability. The recent ACC/AHA guidelines (33) recommend SAVR in patients ≥65 with AS while TAVR should be considered in patients >65 years of age. Utilizing aortic annular/root enlargement, SAVR not only can provide a much larger valve to avoid PPM but also can protect the native aortic root and could potentially decrease the incidence of complete heart block and need for permanent pacemaker during TAVR by constraining the TAVR valve in the SAVR valve (2, 34–36). TAVR in TAVR, also called redo-TAVR may be feasible, but there are concerns for prohibitive coronary obstruction and sinus sequestration with the excursion of the index TAVR leaflets (37–40). Alternatively, SAVR after TAVR has been associated with a high operative mortality, up to 20% (41, 42), which is much higher than redo SAVR (43, 44). In summary, in the post-TAVR era, both SAVR and TAVR can be considered for lifetime management in patients with aortic valve disease. The Heart Team approach remains crucial for appropriate patient selection. At a high-volume academic center, the Heart Team discussion regarding the management of aortic valve disease is patient-centered and while shared decision making is emphasized, SAVR is and will continue to be an excellent option with low risk of morbidity and mortality and the option of future ViV TAVR, especially in the younger patient.

There are considerations when interpreting the results of this study. This study is limited by the retrospective design and single-center experience. In addition, our institution is a quaternary care institution with a structural heart center and may not translate to all facilities. And finally, this study is based on STS data elements and therefore is limited by lack of granularity and of mid- and long-term data regarding re-interventions and survival.

## Conclusion

Isolated SAVR in the post-TAVR era (2012–2020) can be performed with excellent postoperative outcomes and low mortality. SAVR continues to evolve as is evidenced by increased implantation of bioprosthetic valves as well as larger valves to enable future valve-in-valve TAVR. SAVR remains a critical component in the lifetime management of aortic valve disease.

## Data Availability

The raw data supporting the conclusions of this article will be made available by the authors, without undue reservation.
